# Translocating lipopolysaccharide correlates with the severity of enterovirus A71-induced HFMD by promoting pro-inflammation and viral IRES activity

**DOI:** 10.1186/s13099-021-00465-x

**Published:** 2021-11-22

**Authors:** Yuya Wang, Kena Dan, Xiaoling Xue, Xiongbo Yang, Xujiao Feng, Qingqing Yang, Jing Yang, Bangtao Chen

**Affiliations:** 1 Department of Obstetrics and Gynecology, The Third Affiliated Hospital of Chongqing Medical University, Chongqing, 401120 China; 2 Department of Dermatology, The Third Affiliated Hospital of Chongqing Medical University, Chongqing, 401120 China; 3 Department of Hematology, The Third Affiliated Hospital of Chongqing Medical University, Chongqing, 401120 China; 4 Department of Dermatology, Chongqing University Three Gorges Hospital, Chongqing, 404100 China; 5 Department of Infectious Diseases, Heping Hospital Affiliated to Changzhi Medical College, Changzhi, 046000 China

**Keywords:** Hand, foot, and mouth disease, *Enterovirus* A71, 2A protease (2A^pro^), Lipopolysaccharide, Inflammation, Internal ribosomal entry site (IRES)

## Abstract

**Background:**

The increase of inflammation-inducing enterobacteria was recently observed in severe hand, foot, and mouth disease (HFMD) caused by *Enterovirus* A71 (EV-A71). This study aimed to verify the occurrence of bacterial translocation (BT) and further explore the contributory role of BT to severity of EV-A71-mediated HFMD cases.

**Methods:**

Serum specimens from 65 mild and 65 severe EV-A71-associated HFMD cases and 65 healthy children were collected. EV-A71 VP1 in serum, inflammatory mediators including C-reactive protein, IL-1β, IL-6, interferon-γ and tumor necrosis factor-α, BT related biomarkers including Claudin-3, intestinal fatty acid binding protein, lipopolysaccharide (LPS), soluble CD14 (sCD14) and endotoxin core antibody were measured by ELISA. Bacterial DNA (BactDNA) fragments were quantified by quantified PCR (qPCR). Rhabdomyosarcoma (RD) or SH-SY5Y cells, infected with LPS-pre-incubated EV-A71 or transfected with plasmid containing viral 2A^pro^ or mRNA containing viral internal ribosomal entry site (IRES), were post-treated with or without LPS in vitro. EV-A71 RNA and viral or cellular proteins were determined by qPCR and western blot, respectively.

**Results:**

Compared to mild HFMD patients, remarkably higher inflammatory mediators as well as BT-related biomarkers except BactDNA were observed in severe HFMD cases (all *P* < 0.05). In severe HFMD group, circulating concentrations of LPS and sCD14 showed statistical correlations with inflammation indices (all *P* < 0.05), serum levels of EV-A71 VP1 were found to be positively correlated with serum LPS (*r* = 0.341, *P* = 0.005) and serum sCD14 (*r* = 0.458, *P* < 0.001). In vitro, EV-A71 attachment and internalization were only slightly promoted by LPS pre-incubation; however, EV-A71 proliferation and viral 2A^pro^-mediated IRES activity were significantly accelerated by LPS post-treatment.

**Conclusions:**

Our results collectively indicate that gut-derived translocating LPS contributes to the severity of EV-A71-induced HFMD by driving inflammatory response and viral proliferation via viral 2A^pro^-mediated IRES.

**Supplementary Information:**

The online version contains supplementary material available at 10.1186/s13099-021-00465-x.

## Background

*Enterovirus* A71 (EV-A71) is well known to be the major etiological culprit causing hand, foot, and mouth disease (HFMD) in children aged five and below. EV-A71-associated HFMD generally presents as a self-limiting illness. However, some patients may rapidly develop neurological complications and cardiopulmonary disorders that occasionally even cause death. In 1969, Schmidt et al. [1] isolated the first strain of EV-A71 from the stool samples of children with disease of the central nervous system in California, USA. Since then, several outbreaks of EV-A71 infection have been reported across the Asia–Pacific region. In China, it caused the death of 479 children during 2008–2009 and more than 1 million cases per year have been monitored since 2008 [2, 3]. Note worthily, three inactivated monovalent EV-A71 vaccines were licensed in China in 2016; however, the vaccines are only available in the private market in China and the vaccines’ effectiveness against severe HFMD remains yet unknown [4]. And to date, few established antiviral therapies are available for severe EV-A71 infection. Collectively, EV-A71-associated HFMD (especially the severe conditions) still pose a growing global public health and economic concern in affected areas.

EV-A71 is a non-enveloped, positive-sense, single-stranded RNA virus that belongs to genus *Enterovirus* in the family *Picornaviridae*. Structurally, the icosahedral virus particle harbors a RNA genome of approximately 7.4 kb in size with two open reading frames, which is flanked by a highly structured 5′-untranslated region (5′UTR) and a 3′UTR with a poly (A) tail [5]. EV-A71 5′UTR contains a type I internal ribosomal entry site (IRES) mediating initiation of viral proteins translation. By IRES-driven translation, four structural viral proteins (VP1–VP4) and seven non-structural viral proteins (2A–2C and 3A–3D) are finally synthesized with the *cis*-cleavage actions of viral proteases (2A^pro^ and 3C^pro^) [6].

For the survival of EV-A71 in host, viral proteases are the most important promoters for evading host’s antiviral innate immunity by hijacking host cell cap-dependent translation via hydrolysis of eukaryotic initiation factor 4GI (eIF4GI) and other cellular proteins [7]. Although the exact pathogenesis of severe HFMD caused by EV-A71 has not been fully elucidated, increasing evidence have shown that inflammatory mediators including interleukin-1β (IL-1β), IL-6, interferon-gamma (IFN-γ), C-reactive protein (CRP), tumor necrosis factor-α (TNF-α), etc. contribute to the development and severity of EV-A71-associated HFMD in Children [8, 9]. The potential mechanism of systemic inflammation activation accompanied by EV-A71 infection has not yet been determined; a recent study preliminarily demonstrated that the up-regulation of inflammation-inducing enterobacteria may be the prevailing cause for severity of HFMD [10].

Enteric dysbacteriosis along with increased intestinal mucosal permeability will result in a higher translocation rate of microbial immunogenic components from the gut into the circulatory system, which is the so-called bacterial translocation (BT). Serological indicators of leaky gut including Claudin-3 and intestinal fatty acid binding protein (I-FABP), bacterial components including lipopolysaccharide (LPS) and bacterial DNA (BactDNA), products to LPS challenge including soluble CD14 (sCD14) and endotoxin core antibody (EndoCAb) are usually applied to evaluate BT [11–17]. Clinically, BT was proved to be associated with systemic inflammation in patients with cirrhosis [13], psoriasis [14], inflammatory bowel disease [15], hepatitis virus and human immunodeficiency virus infection [16, 17]. However, occurrence of leaky gut-related BT and its association with exacerbation of inflammatory response in HFMD children are poorly investigated.

Taken together, we hypothesize that pro-inflammation cytokines characterize the severity of HFMD and increased intestinal permeability-caused BT is one of main culprits for tuning process of inflammation. With regard to this, in present study, we focused on the correlations between leaky gut-related BT and inflammation-driven severity of HFMD, and further assessed the possible mechanism of BT in EV-A71 infection, in the hope of providing more convincing evidence for BT-derived inflammatory pathogenesis of HFMD deterioration.

## Materials and methods

### Subjects

This study was approved by and carried out under the guidelines of the Ethics Committee of Heping Hospital affiliated to Changzhi Medical College. Before enrollment, informed consent was obtained from the parents/guardians of all the recruited children. Total 130 EV-A71-induced HFMD patients (65 mild cases and 65 severe cases) and 65 age- and gender-matched healthy children were collected during 2015 to 2018 in Heping Hospital. All the patients were etiologically confirmed by EV-A71 RNA detection in stool or throat swabs. According to the Chinese guidelines for the diagnosis and treatment of HFMD (2018 edition) [18], severe HFMD cases were clinically diagnosed if they experienced any neurological complications and/or cardiopulmonary complications. Children with other comorbidities such as juvenile idiopathic arthritis etc. or medications such as systemic anti-inflammation are excluded.

### Laboratory examination

Peripheral blood samples were collected from all the subjects. Blood cell count and liver function were routinely examined. The protein levels of indicators assessed by enzyme-linked immunosorbent assay (ELISA) in present study involved CRP (#E007462, 3ABio), IL-1β (#E001772, 3ABio), IL-6 (#E000482, 3ABio), IFN-γ (#C608-01, GenStar), TNF-α (#489204, Cayman), LPS (#DG11072H, Dogesce), I-FABP (#DFBP20, R&D Systems), Claudin-3 (#abx250611, Abbexa), sCD14 (#DC140, R&D Systems) and EndoCAb (#E013362, 3ABio). Human EV-A71 VP1 protein ELISA kit (#MM-13481H2, MeiMian) was applied to detect the protein level of EV-A71 VP1 from blood serum. Assays were performed according to the manufacturer’s specifications and the detection limits were in line with the manufacturer’s instructions. All the plates were read by the I Mark™ Micro plate Reader (BIO-RAD).

### Cell culture, virus infection, transfection, stimulation and luciferase assay

Human rhabdomyosarcoma (RD) cells (ATCC® CCL-136) and SH-SY5Y cells (Procell, #CL-0208) were maintained in Dulbecco’s modified Eagle’s medium (DMEM, Gibco) containing 10% fetal bovine serum (FBS, Hyclone) with 100 U/mL penicillin and 100 μg/mL streptomycin. The cells were infected with BrCr strain EV-A71 (ATCC® VR-1432™) or neurotropic strain EV-A71 (Xiangyang-Hubei-09, GenBank accession no. JN230523.1) at the multiplicity of infection (MOI) of 2 or transfected with N-terminal GFP-tagged EV-A71 2A expression plasmid and/or bi-cistronic reporter plasmid containing Cap-Rluc-vIRES-Fluc. Plasmid construction, transcription in vitro and transfection using Lipofectamine 2000 reagent (Life Technologies) were done as previously described [19]. LPS (#tlrl-peklps, InvivoGen) pre-incubation or post-treatment were specified in figure legends. Observation of cell morphology was performed with microscope. The detail information about cell culture, virus preparation and virus infection and luciferase assay referred to other reports [19].

### Western blot and antibodies

The RD or SH-SY5Y whole-cell lysates were prepared by lysing with RIPA buffer and western blot was performed as Wang et al. [20] described. Anti-EV-A71 VP1 (#PAB7631-D01P) and anti-β-actin (#BE0021-1000) were obtained from Abnova and EASYBIO, respectively. Anti-ERK1/2 (#9102), anti-phospho-ERK1/2 (#9101) and anti-eIF4GI (#2858) were purchased from Cell Signaling Technology. The target protein and β-actin were detected with anti-rabbit or mouse secondary antibody conjugated with horseradish peroxidase (#BE0103-100 and # BE0108-100, EASYBIO). Specific bands were visualized with enhanced chemiluminescent substrate (ECL) and density of visualized bands was conducted using Quantity One software. Each immunoblot assay was carried out at least three times and one of them was presented.

### Quantification of BactDNA fragments and EV-A71 RNA

Quantification of BactDNA fragments was performed as previously described [21]. To avoid potentially bacterial contamination of molecular biology reagents, all specimens were processed in airflow chambers by the same investigator and all tubes were never exposed to free air. To remove potentially confounding 16S rDNA contamination, 6 tubes of prepared DEPC water were set as negative controls and the processes of water from DNA extraction to quantitative PCR (qPCR) were completely synchronized with those of blood.

Genomic DNA was isolated from a total of 200 μL of serum with QIAmp DNA Blood Minikit (Qiagen, Hilden, Germany) according to the manufacturer's instructions and DNA was eluted in a 100 μL final volume. BactDNA levels were determined by qPCR in a 20 μL amplification reaction with forward primer (5′-AGAGGGTGATCGGCCACA-3′) and reverse primer (5′-TGCTGCCTCCCGTAGGAGT-3′), the universal eubacterial primers of a conserved region of 16S rDNA gene. The amplification conditions for the 59 base pairs of DNA fragment were 95 °C for 10 min, followed by 45 cycles at 95 °C for 15 s and 60 °C for 60 s. Each sample was amplified in triplicate and the BactDNA content was calculated according to a standard curve that generated from serial dilutions of plasmid DNA containing known copy numbers of the template. The final circulating BactDNA concentration was calculated by subtracting proportion of 16S rDNA copies/μL detected in water controls from those in blood.

Quantification of EV-A71 RNA was performed as previously described [22].

### Statistical analyses

Data were analyzed using IBM SPSS software (version 17.0, SPSS Inc., China) and expressed as the mean (M) ± standard deviation (SD) or number (%). Normal distribution of raw data were confirmed by Kolmogorov–Smirnov tests. There were no outliers in continuous data by inspection of related boxplots. For comparison of demographic information and clinical characteristics at baseline among groups, Fisher’s exact Chi-square test or one-way analysis of variance (ANOVA) were conducted except specification. Analysis of covariance (ANCOVA) controlling for age and gender was used to analyze cytokines and bacterial measures among the three groups, and Bonferroni’s multiple comparison test that can calculate the corrected statistical significance for multiple comparisons was performed for post-hoc analysis of pairwise comparisons. Partial correlation analysis controlling for age, gender and disease course was used to determine the relationship between bacterial measures and inflammation cytokines or serum viral proteins. All the tests were two-sided. A *P*-value < 0.05 was accepted as the cutoff for statistical significance.

## Results

### General characteristics of the participants

The participants’ characteristics are summarized in Table 1. Among patients, children in the severe group were much younger (27.57 ± 15.53 months) than who in the mild group (39.75 ± 23.81 months, *P* < 0.001), moreover, severe cases were more prone to have high body temperature, increased heart rate, elevated counts of white blood cells, monocyte and platelet in blood (all *P* < 0.05). Distributions of typical rashes in severe patients resembled those in mild patients, whereas erythematous and/or papulovesicular eruptions (atypical rashes) more frequently occurred in the mild (10.77% vs. 1.54%, *P* < 0.05) and herpangina was more common in the severe (64.62% vs. 21.54%, *P* < 0.01). Of the severe patients, the most common complication was neurological dysfunction (78.46%), followed by pulmonary disorders (29.23%) and cardiovascular disorders (6.15%). Furthermore, there were no differences in aspects of heart rate, body temperature and laboratory results between healthy children and mild cases (all *P* > 0.05).

**Table 1:** Clinic and laboratory characteristics of healthy subjects and HFMD patients

Items	Healthy control (n = 65)	Mild HFMD (n = 65)	Severe HFMD (n = 65)
Gender (female/male)	28/37	29/36	28/37
Age (months)	41.42 ± 15.44	39.75 ± 23.81	27.57 ± 15.53***
EV-A71 positive, n (%)	–	65 (100)	65 (100)
Typical rashes^a^, n (%)	–	55 (84.62)	56 (86.15)
Hands	–	50 (76.92)	56 (86.15)
Feet	–	49 (75.38)	53 (81.54)
Mouth	–	42 (64.62)	50 (76.92)
Buttock	–	19 (29.23)	26 (40.00)
Atypical rashes^b^, n (%)	–	7 (10.77)	1 (1.54)*
Herpangina, n (%)	–	14 (21.54)	42 (64.62)**
Cardiovascular disorders, n (%)	–	–	4 (6.15)
Neurological disorders, n (%)	–	–	51 (78.46)
Pulmonary disorders, n (%)	–	–	19 (29.23)
Heart rate (/min)	123.5 ± 15.23	127.12 ± 18.37	144.62 ± 18.04***
Body temperature (°C)	37.45 ± 0.85	37.45 ± 0.37	38.38 ± 0.62***
WBC count (10^9^/L)	7.43 ± 1.66	8.78 ± 1.78	12.01 ± 3.42***
Lymphocyte count (10^9^/L)	3.22 ± 1.16	3.65 ± 1.40	4.23 ± 3.28
Monocyte count (10^9^/L)	0.54 ± 0.25	0.57 ± 0.24	0.81 ± 0.62**
Platelet count (10^9^/L)	239.37 ± 53.62	254.92 ± 49.27	277.37 ± 57.85**
CK (U/L)	102.37 ± 47.83	106.64 ± 52.45	112.23 ± 104.35
ALT (IU/L)	22.20 ± 6.15	23.37 ± 8.53	25.53 ± 13.28
AST (IU/L)	32.53 ± 15.36	33.53 ± 17.63	36.35 ± 20.25
LDH (U/L)	261.24 ± 46.53	276.25 ± 54.53	292.43 ± 78.53
Sampling time (d)^c^	–	2.51 ± 0.82	2.77 ± 0.91

The data were presented as number of patients (%) or M ± SD

*WBC* white blood cells, *CK* creatine kinase, *AST* aspartate transaminase, *ALT* alanine aminotransferase, *LDH* lactate dehydrogenase

Compared with mild HFMD group, *P < 0.05, **P < 0.01, ***P < 0.001

^a^Maculo-papular and/or vesicular rashes

^b^Erythematous and/or papulovesicular eruptions

^c^Timing of serum sampling after onset of HFMD

### Pro-inflammation phenotype dominates in HFMD cases

**Fig. 1:**
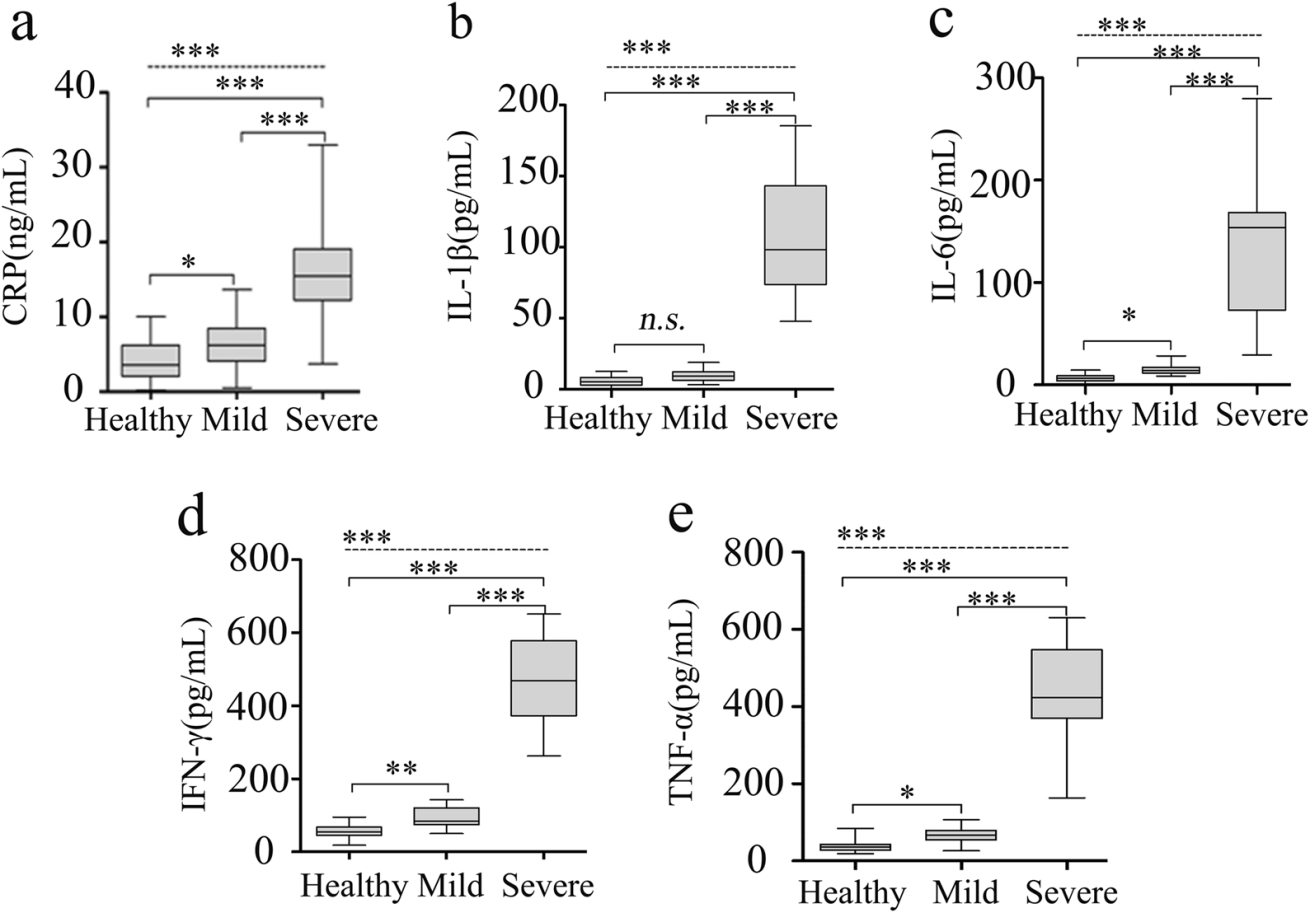
Differentially displayed cytokines in the three groups. *CRP* C-reactive protein, *IL* interleukin, *TNF* tumor necrosis factor, *IFN* interferon. Data were presented as boxplots. In post-hoc analysis using Bonferroni’s multiple comparison test, n.s. > 0.05, *P < 0.05, **P < 0.01, ***P < 0.001; analysis using ANCOVA

As shown in Fig. 1, the results of ANCOVA analysis displayed that there were statistically significant differences between the healthy group, the mild and severe HFMD groups in terms of CRP (*F* = 138.5, *P* < 0.001), IL-1β (*F* = 361.4, *P* < 0.001), IL-6 (*F* = 276.1, *P* < 0.001), IFN-γ (*F* = 730.9, *P* < 0.001) and TNF-α (*F* = 832.4, *P* < 0.001). Further, post-hoc analysis using Bonferroni’s multiple comparison test found that serum levels of the inflammatory biomarkers dramatically increased approximately 3- to 8-times on average in the severe group in comparison with the mild group (all *P* < 0.001), while the protein levels of sera CRP, IL-6, IFN-γ and TNF-α in mild HFMD group were only one- to twofold higher as compared to health control group (all *P* < 0.05). These results demonstrate and verify the existence of systemic pro-inflammation in EV-A71-associated HFMD cases, especially in the severe patients.

### BT occurs in severe HFMD cases

**Fig. 2:**
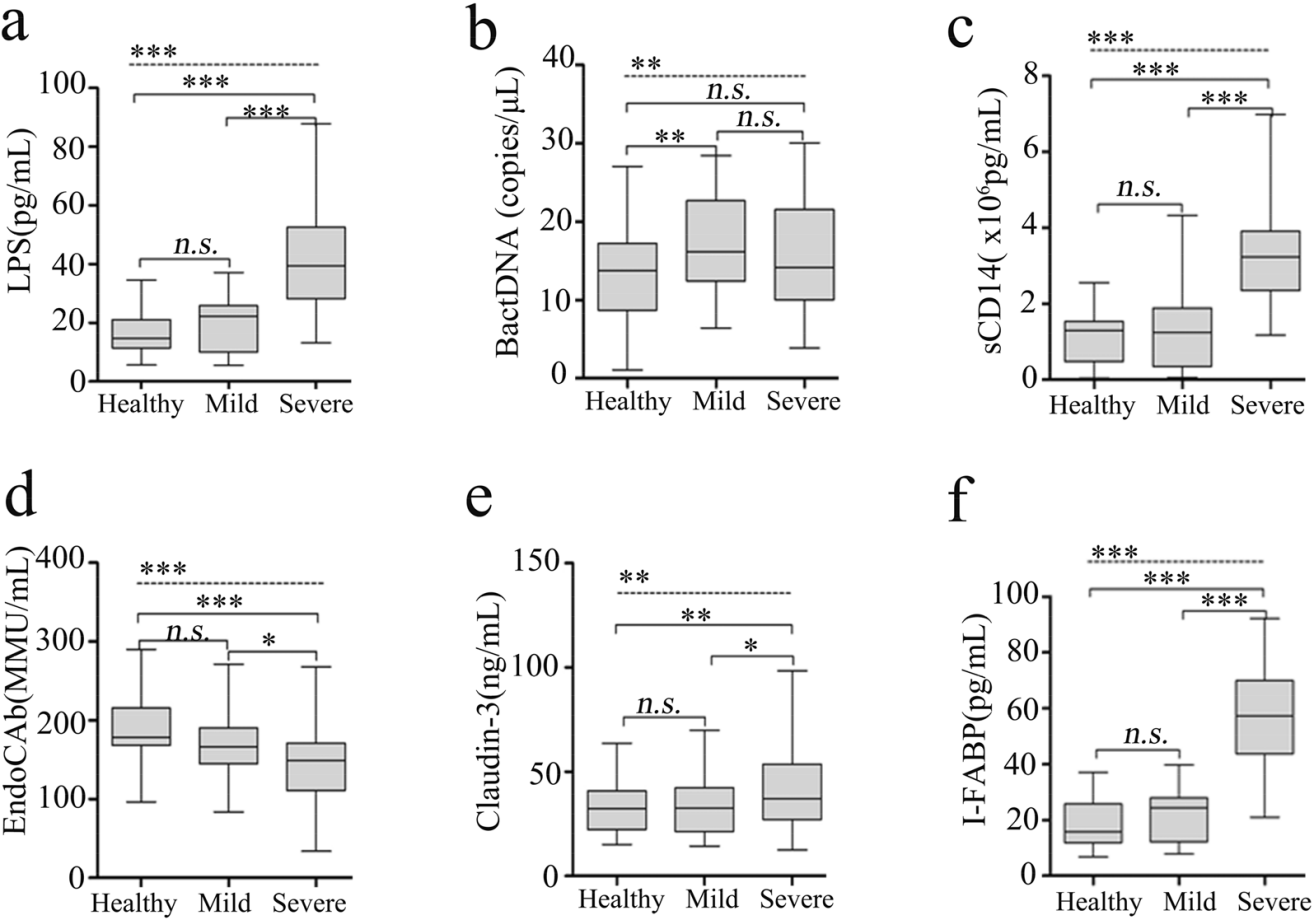
BT-related biomarkers among groups of HFMD and healthy control. *LPS* lipopolysaccharide, *BactDNA* bacterial DNA, *sCD14* soluble CD14, *EndoCAb* endotoxin core antibody, *I-FABP* intestinal fatty acid-binding protein. Data were presented as boxplots. In post-hoc analysis using Bonferroni’s multiple comparison test, n.s. > 0.05, *P < 0.05, ***P < 0.001; analysis using ANCOVA

Next, related serum markers of BT were measured in all the subjects (Fig. 2). Regarding indices of bacterial components (LPS and BactDNA), LPS-response products (sCD14 and EndoCAb), and “leaky gut” (Claudin-3 and I-FABP), statistically significant differences between the three groups were all observed (all *P* < 0.01) from ANCOVA analysis results. Post-hoc analysis showed that only BactDNA titers (17.25 ± 5.78 vs. 13.33 ± 6.97 copies/μL, *P* < 0.01) was moderately increased in the mild HFMD group than healthy control group, while serum concentrations of LPS (41.48 ± 16.78 vs. 19.44 ± 8.81 pg/mL, *P* < 0.001), sCD14 (3.32 ± 1.35 vs. 1.47 ± 1.12 × 10^6^ pg/mL, *P* < 0.001), Claudin-3 (42.53 ± 20.48 vs. 34.39 ± 13.97 ng/mL, *P* < 0.05) and I-FABP (57.16 ± 18.35 vs. 21.51 ± 8.89 pg/mL, *P* < 0.001) were significantly higher, EndoCAb concentration (143.78 ± 52.11 vs. 167.65 ± 39.77 MMU/mL, *P* < 0.05) was remarkably lower in the severe group than the mild group. These data indicate the presence of “leaky gut” and potential BT from intestine in severe HFMD cases.

### LPS positively correlates with inflammation severity and serum viral protein

**Fig. 3:**
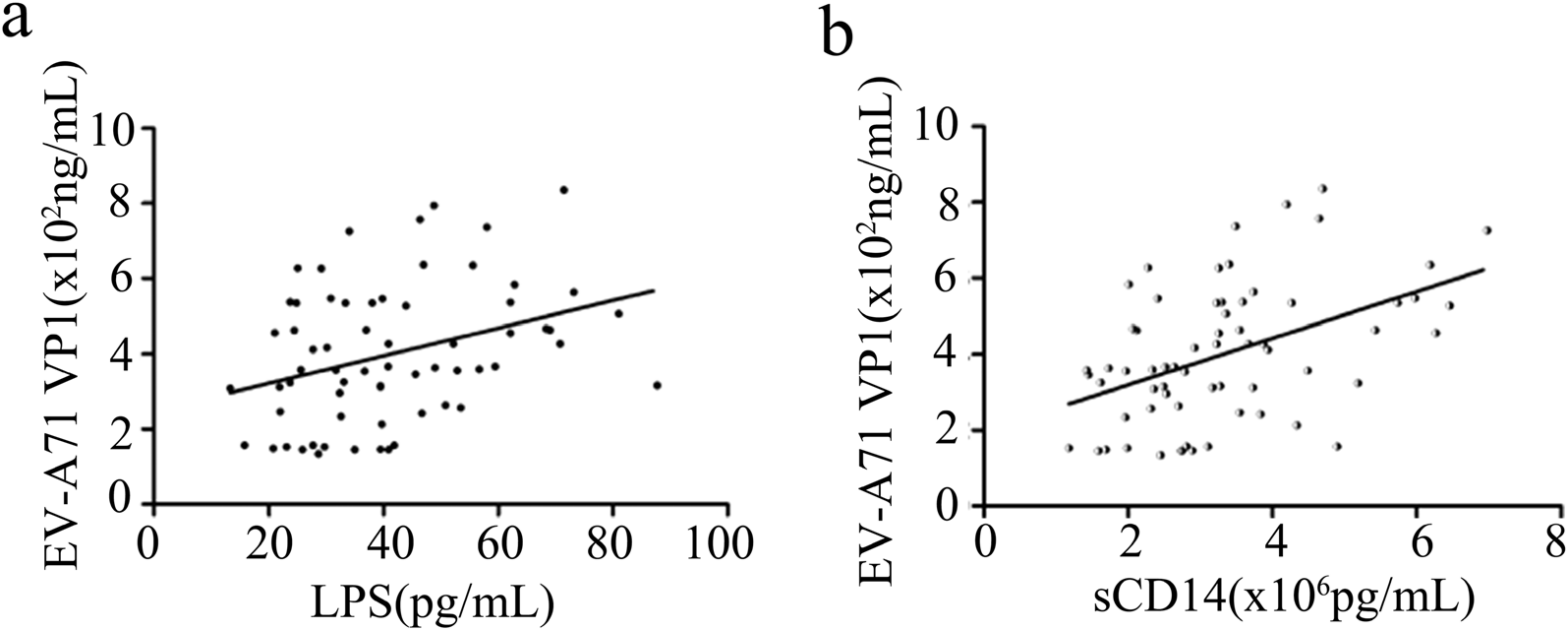
Partial correlation analysis of serum protein levels of EV-A71 VP1 and LPS (**a**) or sCD14 (**b**) in severe HFMD cases

In the severe group (Table 2), circulating concentration of LPS was further found to be positively correlated with all the quantified inflammatory mediators (*P* < 0.05 for all variables); sCD14 was positively associated with CRP (*P* = 0.041), IL-1β (*P* = 0.001), IL-6 (*P* = 0.004) and IFN-γ (*P* = 0.019) after controlling potential confounders. Pro-inflammation was well-proved to facilitate viral replication in vivo and in vitro. In severe HFMD cases, serum protein levels of EV-A71 VP1 determined by ELISA were found to be positively correlated with serum LPS (*r* = 0.341, *P* = 0.005, Fig. 3a) and serum sCD14 (*r* = 0.458, *P* < 0.001, Fig. 3b), respectively. These data imply the link that circulating LPS from BT, as well as LPS responded sCD14, might be the important cause synergistically leading to the higher levels of pro-inflammation mediators and viral proteins observed in severe HFMD patients.

**Table 2:** Correlation between BT and inflammation in severe HFMD patients

Cytokines	CRP		IL-1β		IL-6		IFN-γ		TNF-α	
	*r*	*P*	*r*	*P*	*r*	*P*	*r*	*P*	*r*	*P*
LPS	0.323	0.039*	0.470	0.002*	0.621	0.007*	0.319	0.041*	0.652	0.001*
BactDNA	0.122	0.341	0.237	0.037*	0.258	0.633	0.053	0.346	0.324	0.092
I-FABP	0.072	0.098	0.156	0.481	0.217	0.093	0.143	0.071	0.264	0.271
Claudin-3	0.051	0.365	0.224	0.472	0.232	0.053	0.204	0.094	0.044	0.431
sCD14	0.672	0.041*	0.534	0.001*	0.648	0.004*	0.513	0.019*	0.837	0.052
EndoCAb	0.155	0.362	0.243	0.129	0.047	0.325	0.474	0.061	0.235	0.353

*P < 0.05. Analyses using partial correlation analysis

### LPS pre-incubation slightly promote EV-A71 attachment and internalization

**Fig. 4:**
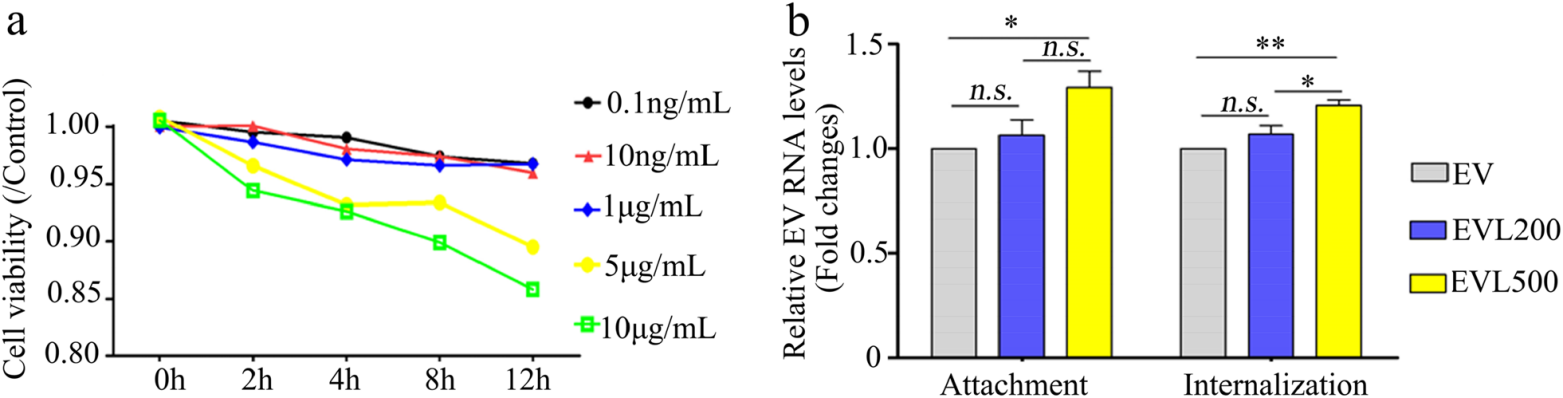
LPS pre-incubation on EV-A71 attachment and internalization. RD cells were treated with 0.1 ng/mL to 10 μg/mL LPS for 0 to 12 h followed by cell viability assessment using CCK8 (**a**). 2 MOI of EV-A71 were pre-incubated with 200 ng/mL or 500 ng/mL LPS at 37 °C for 2 h, then the virus were used to infect RD cells with binding buffer on ice. 1 h later, the cells were washed (attachment assessment) or/and cultured at 37 °C for another 1 h and then treated with trypsin (internalization assessment). Viral RNA was extracted by commercial kit and EV-A71 RNA was determined by qPCR (**b**). Data were showed as M ± SD and *student’s t* test was used for comparisons, n.s. > 0.05, *P < 0.05, **P < 0.01

To explore the effect of circulating LPS on EV-A71 infection, EV-A71 Strain BrCr was used to infect RD cells in vitro after pre-incubating the virus with *E-coli K12*-derived LPS at 37 °C for 2 h. Firstly, cytotoxicity of LPS to RD cells was determined and results found that LPS at less than 1 μg/mL was not toxic to the cells (Fig. 4a). As Fig. 4 showed, only slightly increased levels of EV-A71 RNA on cell surface (relative multiple: 1.29 ± 0.13, *P* < 0.05) and that entering the cell (relative multiple: 1.20 ± 0.06, *P* < 0.01) were only observed in 500 ng/mL LPS treatment group in comparison with mock treatment group, which indicates that LPS pre-incubation only slightly facilitate EV-A71 infection at steps of viral attachment and internalization.

### LPS accelerate EV-A71 replication by promoting viral 2A^pro^-mediated IRES activity

**Fig. 5:**
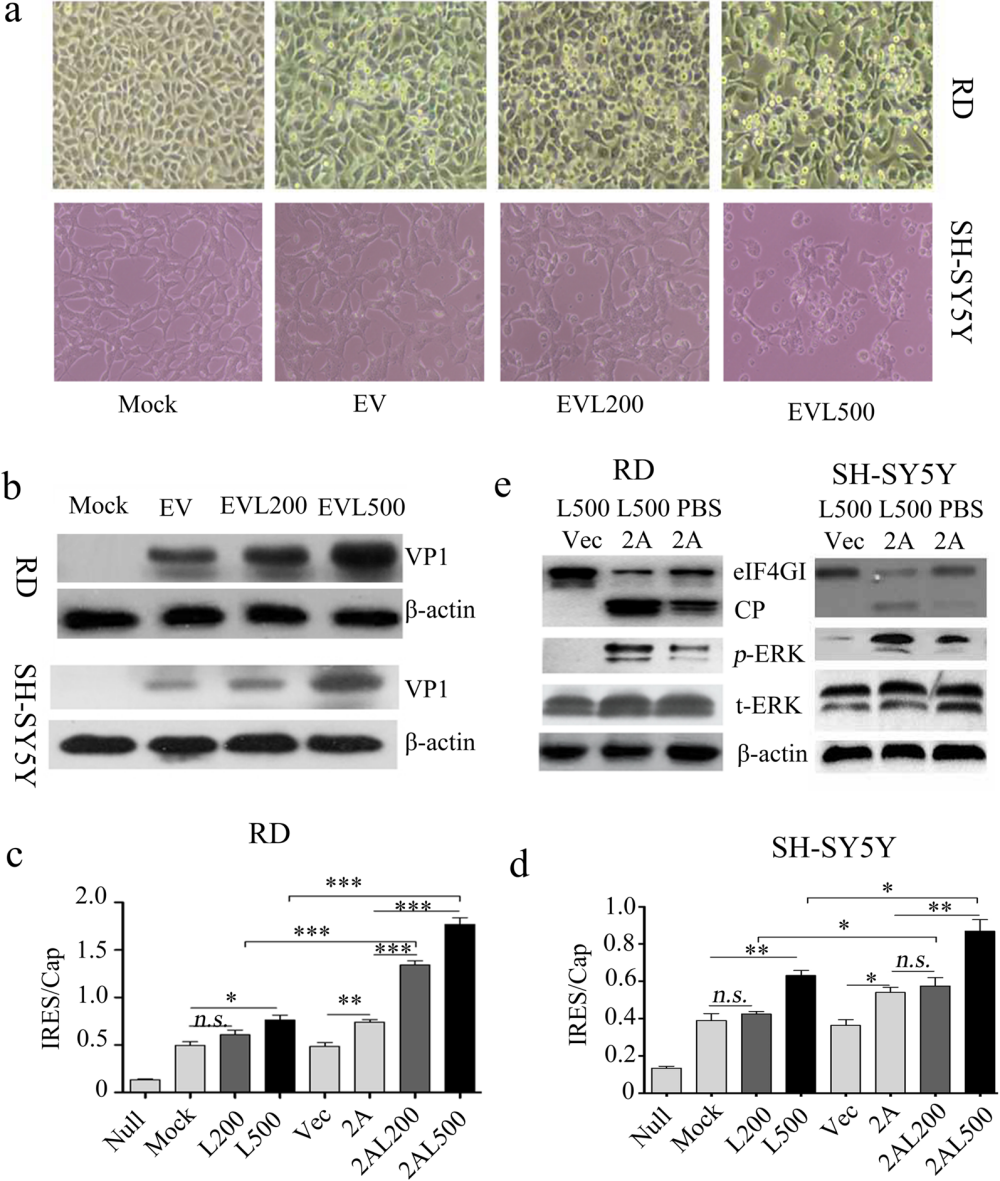
The effect of LPS post-treatment on proliferation of EV-A71 in vitro. RD cells and SH-SY5Y cells were infected with BrCr Strain EV-A71and neurotropic Strain EV-A71 at an MOI of 2, respectively. 2 h later, the cells were treated with LPS at the concentration of 200 ng/mL or 500 ng/mL, respectively. At 12 h post infection, photomicrographs were taken (original magnification, ×100) (**a**), the protein levels of viral VP1 in cell lysates were measured by western blot (**b**). RD cells or SH-SY5Y cells were pre-transfected with p-EGFP-Vector (Vec, 2 μg/well, 6-well plate) or p-EGFP-2A (2A, 2 μg/well, 6-well plate), respectively. Subsequently, 12 h later, the cells were re-transfected with Cap-Rluc-vIRES-Fluc mRNA (100 ng/well, 96-well plate). 4 h later, the cells were treated with 200 ng/mL or 500 ng/mL LPS for another 12 h. The intensities of Fluc and Rluc were detected as described in MMs. The results (Rluc/Fluc) indicate the M ± SD of three independent experiments (**c**, **d**). RD cells or SH-SY5Y cells were pre-transfected with p-EGFP-Vector (Vec) or p-EGFP-2A (2A), respectively. 12 h later, the cells were treated with 500 ng/mL LPS for another 12 h. The cell lysates were used for the protein detection of eIF4GI, phosphorylated-ERK (p-ERK) and total ERK (t-ERK) by western blot (**e**). EV, EV-A71; EVL200, EV-A71 + 200 ng/mL LPS; EVL500, EV-A71 + 500 ng/mL LPS. 2AL200, 2A + 200 ng/mL LPS; 2AL500, 2A + 500 ng/mL LPS. Statistical difference was determined by *student’s t* test. *n.s.* > 0.05, *P < 0.05, **P < 0.01, ***P < 0.001

**Fig. 6:**
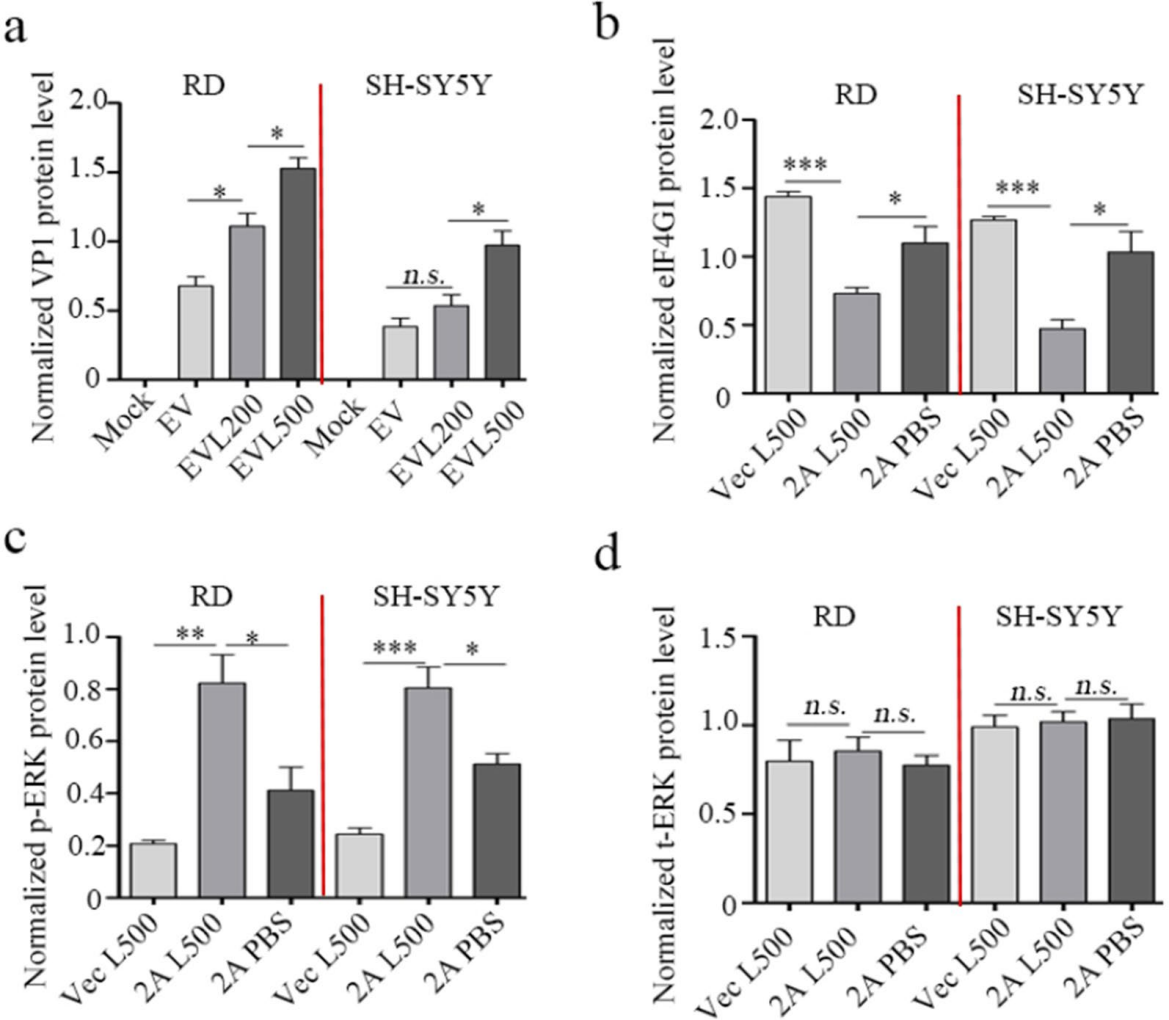
The densitometric analysis bar diagram of western blot results. Densitometric analysis of the western blot results in Fig. 5b, e was conducted using Quantity One software. VP1 (**a**), eIF4GI (**b**), p-ERK (**c**) and t-ERK (**d**) expression level was normalized by β-actin expression level. Columns represent the mean from three independent experiments and bars represent standard deviations. Statistical difference was determined by *student’s t* test. *n.s.* > 0.05, *P < 0.05, **P < 0.01, ***P < 0.001

The effect of LPS post-treatment on EV-A71 proliferation in vitro was further examined. The cytotoxicity of LPS at a concentration of less than 1 μg/mL on SH-SY5Y cells was similar to what observed in setting with RD cells used (Additional file 1: Fig. S1). Compared to virus-infected cells without LPS challenge, 500 ng/mL LPS treatments dramatically promoted the occurrence of cytopathic effect (CPE) of RD cells induced by BrCr Strain EV-A71 infection and CPE of SH-SY5Y cells induced by neurotropic Strain EV-A71 infection (Fig. 5a), and increased the expressions of intracellular VP1 in the two conditions (Figs. 5b and 6a). Because the synthesis of enterovirus protein is mediated by viral 2A^pro^-driven viral IRES, effect of LPS post-treatment on viral IRES was assessed. As Fig. 5c, d presented, overexpression of viral 2A^pro^ or treatment with 500 ng/mL LPS moderately promoted IRES activity (*P* < 0.05 for all variables) compared with mock treatments. However, when compared with 2A^pro^ or LPS treatment, IRES activity was remarkably increased by 200 ng/mL or 500 ng/mL LPS in 2A^pro^-overexpressed RD cells and by 500 ng/mL LPS in 2A^pro^-overexpressed SH-SY5Y cells (*P* < 0.05 for all variables). We previously proved that 2A^pro^-driven viral IRES activity was regulated by cellular phosphorylated extracellular signal-regulated kinase (ERK)-mediated eIF4GI trans-cleavage [23]. Figures 5e and 6b–d showed that 2A^pro^-mediated phosphorylation of ERK and cleavage of eIF4GI were significantly accelerated by 500 ng/mL LPS in RD and SH-SY5Y cells. These data collectively demonstrate that LPS can facilitate EV-A71 replication by promoting viral 2A^pro^-mediated IRES activity, which imply the contributory role of translocating LPS to the severity of EV-A71-induced HFMD.

## Discussion

EV-A71 is generally regarded as the major causative agent for severe HFMD cases with neurological complications. Several independent studies have previously shown a close association between elevated inflammatory mediators and HFMD severity [8, 9, 24–26]. Consistent with that, levels of inflammatory mediators including CRP, IL-6, IFN-γ, IL-1β and TNF-α in mild and severe HFMD cases with EV-A71 infection in present study were moderately and dramatically increased, respectively. Serum IL-6 has been reported to be strongly associated with aseptic meningitis among children with EV-A71-induced HFMD [26]. As is well known, to remove pathogenic microorganisms and protect the tissue from damage, CRP rises sharply in the plasma with IL-6 stimulation. IFN-γ, an imperative contributor of the generation of IFN-γ-inducible protein-10 (IP-10), is responsible for the recruitment of Th1 lymphocyte into the central nerve system during EV-A71 infection [8]. Although functional redundancies of IL-1β and TNF-α have been reported, the supposed pyrogenic role that contributes to the febrile response commonly observed in severe HFMD patients would be more crucial [9]. Actually, the increases in production of these inflammatory mediators were unlikely to be purely due to increased viral replication, they may interact and synergize to induce tissue damage in a sophisticated and coordinated network [8]. Previous profiling studies mainly explored the changes in inflammatory cytokines and potential cytokines for predicting the severity and criticality of HFMD. In present study, the involvement of inflammation response was further verified, more than that, we were particularly interested in the potential causes for systemic inflammation activation and we focused on the BT in EV-A71-associated HFMD individuals, which had never been investigated before.

BT is the passage of bacterium and/or bacterial products from the gut lumen into the organism in absence of bacteremia. In this process, increased intestinal mucosal permeability is indispensable. The tight junction is universally demonstrated to be the structural basis for maintaining normal intestinal permeability [27, 28]. Claudin-3 and I-FBPA, the two key components of tight junction, present at high levels in the blood can reliably reflect increased intestinal permeability as they are released into systemic circulation by enterocytes when intestinal epitheliums are compromised.

In present study, the evidence that remarkable increases in serum levels of I-FABP and Cludin-3 only in severe cases links the increased intestinal permeability with the severity of EV-A71-associated HFMD, which has not been reported previously. Correspondingly, the peripheral blood concentration of LPS, but not BactDNA, was significantly higher in severe other than mild cases with EV-A71 infection. Translocating LPS is in fact related to an exacerbation of the inflammatory response [29] and the following correlation analysis also showed that the circulating concentrations of inflammatory mediators had good correlativity with LPS, as well as sCD14. As LPS-specific host response, sCD14 circulates at high levels in the serum and interacts with translocating LPS to stimulate antigen-presenting cells via toll-like receptor 4 (TLR4) signaling [30]. Under bacteria or LPS challenge, vascular endothelial cells and perivascular mast cells have been reported to express abundant TLR4, thus, the inflammatory cytokines are synthesized and secreted [31–33]. Furthermore, decreased host EndoCAb in peripheral blood failed to bind and clear LPS from circulation, which ensures high serum level of LPS for a long time and subsequently maintains systemic inflammation. It is also worth noting that serum BactDNA loads in mild cases with aggression may have little effect on inflammation state given the results from correlation analysis and differential expressions of BactDNA among mild or severe cases. We can only speculate that serum BactDNA loads quantified by qPCR likely underestimate the presence of BactDNA within whole blood and corresponding perturbation of inflammation markers may be transient. Collectively, these findings emphasize that translocating LPS is implicated in EV-A71-induced systemic inflammation responses and argue for a causative relationship between circulating LPS and disease exacerbations.

Most inflammatory cytokines are crucial immune modulators in host-virus interaction. Upon viral infection, the fine-tuning levels of myriad inflammatory mediators usually determine an anti-viral state advantageous to the hosts or a pro-viral state advantageous to the invading viruses. Translocating LPS will undoubtedly aggravate inflammatory response and may correspondingly promote viral propagation. Clinically, LPS, as well as sCD14, was demonstrated to be positively correlated with EV-A71 VP1 loads in serum in present study. Intestinal bacterial surface LPS was uncovered to bind poliovirus (a member of *Enterovirus* genus) and thus enhanced virion stability and cell attachment [34], which may be also exploited by EV-A71 for replication and transmission. In vitro*,* we further demonstrated that EV-A71 attachment and internalization were only slightly promoted by LPS pre-incubation; in contrast to that, EV-A71 proliferation in RD and SH-SY5Y cells were significantly facilitated by LPS post-incubation, which was further proved to be linked with viral 2A^pro^-mediated IRES activity. Apart from the mentioned perspectives, LPS was also proved to stimulate early growth response-1 (EGR1) translocation into the nucleus and the nuclear EGR1 facilitates EV-A71 replication by binding to EV-A71 5′ UTR, a region that contains IRES structure [35].

Unfortunately, at least four limitations exist in our study. First, correlations between these inflammatory mediators and BactDNA in mild patients were not conducted as they were moderately elevated in comparison to the healthy children. Second, we didn’t perform stratified analyses in subgroups of neurological dysfunction or cardio-respiratory disorders, which is partly to blame for the limited enrollment of severe cases. Furthermore, as with all case-controlled clinical studies, present study failed to adequately explain the causal relationship between BT and disease severity, related animal experiments are expected for ethical considerations. Last but not the least, the molecular mechanism by which translocating LPS promotes systemic inflammation and aids viral replication remains to be further investigated.

## Conclusion

Current study mainly verifies the presence of leaky gut-caused bacterial translocation and further correlates translocating LPS to severity of EV-A71-induced HFMD possibly by driving pro-inflammation response and promoting viral 2A^pro^-mediated IRES activity. Collectively, these observations indicate that bacterial translocation may be a novel anti-inflammatory or antiviral therapeutic target for improving disease outcome in severe cases with EV-A71 infection.

## Supplementary Information

**Additional file 1: Figure S1.** The cytotoxicity of LPS on SH-SY5Y cells. SH-SY5Y cells were treated with 200 ng/mL, 500 ng/mL or 1 μg/mL LPS for 12 h followed by cell viability assessment using CCK8.

## Data Availability

All data involved in this study is available upon reasonable request made to the corresponding author.

## Abbreviations

LPS: Lipopolysaccharide
EV-A71: *Enterovirus* A71
HFMD: Hand, foot, and mouth disease
IRES: Internal ribosomal entry site
IFN-γ: Interferon-gamma
CRP: C-reactive protein
TNF-α: Tumor necrosis factor-α
I-FABP: Intestinal fatty acid binding protein
sCD14: Soluble CD14
EndoCAb: Endotoxin core antibody
eIF4GI: Eukaryotic initiation factor 4GI
CPE: Cytopathic effect
ERK: Extracellular signal-regulated kinase
TLR4: Toll-like receptor 4
EGR1: Early growth response-1
