# Supplementary material for: Translocating lipopolysaccharide correlates with the severity of enterovirus A71-induced HFMD by promoting pro-inflammation and viral IRES activity
Source: Gut Pathog. 2021 Nov 22;13:69. doi: 10.1186/s13099-021-00465-x (PMC8607650; doi:10.1186/s13099-021-00465-x)
Supplement: Supplementary file 1 — Additional file 1: Figure S1. The cytotoxicity of LPS on SH-SY5Y cells. SH-SY5Y cells were treated with 200 ng/mL, 500 ng/mL or 1 μg/mL LPS for 12 h followed by cell viability assessment using CCK8. [file 13099_2021_465_MOESM1_ESM.docx]

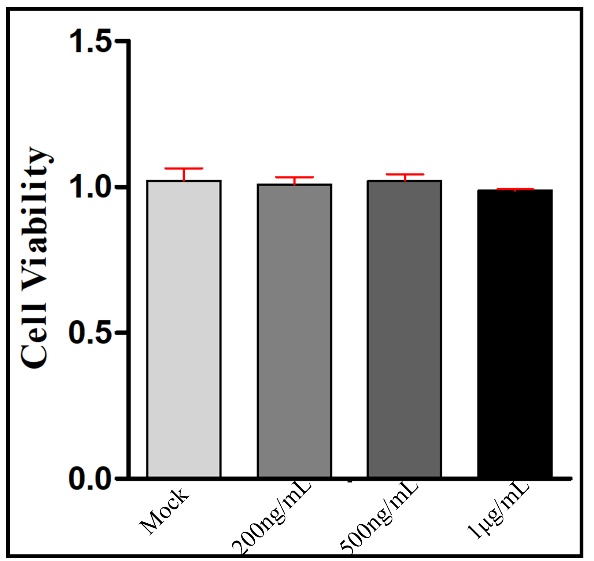


Figure S1. The cytotoxicity of LPS on SH-SY5Y cells. SH-SY5Y cells were treated with 200ng/mL, 500ng/mL or 1μg/mL LPS for 12 hours followed by cell viability assessment using CCK8.
